# What Eye Movements Can Tell about Theory of Mind in a Strategic Game

**DOI:** 10.1371/journal.pone.0045961

**Published:** 2012-09-28

**Authors:** Ben Meijering, Hedderik van Rijn, Niels A. Taatgen, Rineke Verbrugge

**Affiliations:** 1 Department of Artificial Intelligence, University of Groningen, Groningen, The Netherlands; 2 Department of Experimental Psychology, University of Groningen, Groningen, The Netherlands; George Mason University/Krasnow Institute for Advanced Study, United States of America

## Abstract

This study investigates strategies in reasoning about mental states of others, a process that requires theory of mind. It is a first step in studying the cognitive basis of such reasoning, as strategies affect tradeoffs between cognitive resources. Participants were presented with a two-player game that required reasoning about the mental states of the opponent. Game theory literature discerns two candidate strategies that participants could use in this game: either *forward reasoning* or backward reasoning. Forward reasoning proceeds from the first decision point to the last, whereas backward reasoning proceeds in the opposite direction. Backward reasoning is the only optimal strategy, because the optimal outcome is known at each decision point. Nevertheless, we argue that participants prefer forward reasoning because it is similar to causal reasoning. Causal reasoning, in turn, is prevalent in human reasoning. Eye movements were measured to discern between forward and backward progressions of fixations. The observed fixation sequences corresponded best with forward reasoning. Early in games, the probability of observing a forward progression of fixations is higher than the probability of observing a backward progression. Later in games, the probabilities of forward and backward progressions are similar, which seems to imply that participants were either applying backward reasoning or jumping back to previous decision points while applying forward reasoning. Thus, the game-theoretical favorite strategy, backward reasoning, does seem to exist in human reasoning. However, participants preferred the more familiar, practiced, and prevalent strategy: forward reasoning.

## Introduction

Having a theory of mind (ToM) allows us to reason about other people’s mental states, their knowledge, beliefs, desires, and intentions. This ability is helpful in social interactions, especially when our outcomes depend on the actions of others, and vice versa. Many studies have focused on the age at which ToM develops [1], [2], [3], [4], [5], [6], the proficiency of humans and nonhumans in ToM tasks [7], [8], [9], [10], [11], [12], [13], [14], and the brain regions associated with ToM [15], [16], [17]. In contrast, few studies have focused on the cognitive basis of ToM [18], [19]. Consequently, little is known about how inferences about mental states are achieved.

As findings from cognitive neuroscience have shown that participants in ToM tasks employ many brain regions rather than one single “ToM module” [15], [16], [17], [18], ToM reasoning probably consists of multiple serial and concurrent cognitive processes. Cost-benefit tradeoffs between these various resources will most likely have cascading effects on cognitive load [20] and thus ToM reasoning. Both task setting and strategies, in turn, have been shown to affect cost-benefit tradeoffs between cognitive resources [21], [22], [23]. Therefore, the study of strategies and task setting might be an appropriate first step in the study of the cognitive basis of ToM reasoning [24], [25].

**Figure 1:**
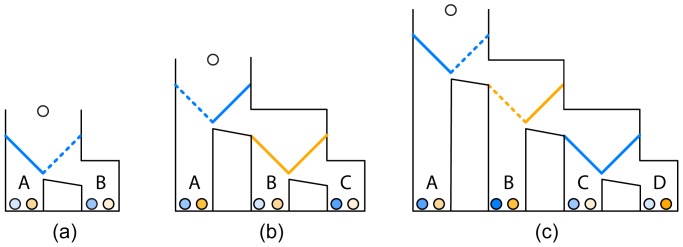
Examples of zero-order (a), first-order (b), and second-order (c) Marble Drop games. Each bin contains a pair of marbles, labeled A to D. For each player, the goal is that the white marble drops into the bin that contains the darkest possible marble of his or her allocated color. In this example, Player 1′s marbles are blue, and Player 2′s marbles are orange. Player 1 controls the blue trapdoors and Player 2 controls the orange trapdoors. The dashed diagonal lines represent the trapdoors that the players should decide to remove to obtain their maximum payoffs in these particular games.

In this study, we investigate the ongoing process of ToM reasoning in a two-player game, referred to as Marble Drop [11], [12], see Figure 1. In this game, a white marble is about to drop, and each player’s goal is that the white marble drops into the bin that contains the darkest possible marble of his or her allocated color. This is commonly known among the players. Both players can remove trapdoors to control the path of the white marble. Marble Drop requires ToM because each player’s outcomes depend on the decisions of the other player.

The example games in Figure 1 are of varying difficulty. With each additional decision point (i.e., set of trapdoors), the required reasoning becomes more complex. The game in Figure 1c is the most difficult, and requires second-order ToM. Below, we provide a possible reasoning scenario to explain how second-order ToM comes into play in this particular game.

By looking at payoff-pairs A to D in the game in Figure 1c, Player 1 will find out that B contains the darkest marble of his allocated color, blue. Player 1 has to ask himself whether that marble is attainable. In other words, Player 1 has to reason about whether Player 2 would remove the left orange trapdoor. Therefore, Player 1 has to look at the orange marbles in B to D to find out that D contains Player 2′s darkest orange marble. ToM reasoning continues with Player 1 asking himself whether Player 2 thinks her orange marble in D is attainable. In other words, Player 1 has to reason about whether Player 2 thinks that he, Player 1, would remove the right blue trapdoor of the rightmost set of trapdoors. Player 1 knows that he would not remove that trapdoor, but that he would remove the left one instead. He also knows that Player 2 is aware of this, as both players are aware of each other’s goals. Therefore, Player 1 knows that Player 2 knows that her darkest orange marble in D is unattainable. Therefore, Player 1 has to go back to the second decision point (i.e., the orange trapdoors). There, Player 2 would compare the orange marbles in B and C and decide to remove the left orange trapdoor, because the orange marble in B is the darkest orange marble that she can still attain. To conclude, Player 1 knows that his darkest blue marble in B is attainable, and will thus remove the right blue trapdoor of the leftmost set of trapdoors.

According to game theory literature there is just one strategy that undoubtedly yields the optimal outcome: reasoning by *backward induction*. We will refer to this strategy simply as backward reasoning. Backward reasoning proceeds from the last decision to be made back to original problem or situation [26]. The last decision in the game in Figure 1c is Player 1′s decision between the blue marbles in payoff-pairs C and D. Player 1 would decide to remove the left trapdoor because C contains the darker blue marble. Backward reasoning would then proceed with the second-to-last decision, which is Player 2′s decision between the orange marbles in payoff-pairs B and C. Player 2 would decide to remove the left orange trapdoor, because B contains the darker orange marble. Backward reasoning stops at the third-to-last decision, which is Player 1′s decision between the blue marbles in payoff-pairs A and B. Player 1 would remove the right blue trapdoor, because B contains the darker blue marble. This scenario shows that backward reasoning is very efficient, because the optimal outcome is known at each decision point. Accordingly, few reasoning steps need to be retained, and working memory load would be small.

Game theory literature discerns another possible strategy, *forward reasoning*, but this strategy is not guaranteed to yield the optimal outcome [24], [27]. Opposite to backward reasoning, the forward reasoning strategy starts at the first decision point in a game and blindly proceeds to the next for as long as higher outcomes are expected to be available at future decision points. A drawback of this strategy is that a player might not recognize the highest attainable outcome and continues the game to future decision points with lower outcomes. However, occasionally forward reasoning yields a quick solution, for example, if the maximum outcome is available at the first decision point.

Even though backward reasoning is the optimal strategy in games such as Marble Drop, it does not seem to be ubiquitous in human reasoning. In contrast, a forward progression seems to be more prevalent, for example in causal reasoning, where causes or decisions *lead* to possible effects. A well-known example of the persistency of causal reasoning is the *fundamental attribution error*, where causal explanations of observed behaviors are often dispositional despite more appropriate situational explanations [28], [29].

Given the prevalence of a forward direction in human reasoning, we expect that forward reasoning might also be a viable candidate strategy in Marble Drop games, even though backward reasoning is the game-theoretical favorite. However, forward reasoning would not always suffice to achieve the optimal outcome in Marble Drop. As explained above, a player might discover, while reasoning forwardly, that he or she unknowingly skipped the highest attainable outcome at a previous decision point. Thus, the player would need to jump back to inspect whether that outcome is indeed attainable. The procedure of jumping back to previous decision points is called *backtracking* [30]. Backtracking superficially resembles backward reasoning, but it differs because jumping back to a previous decision point can be followed up with forward reasoning again. Note that our explanation of the Marble Drop game in Figure 1c followed the procedure of forward reasoning plus backtracking. Forward reasoning plus backtracking is less efficient than backward reasoning, because (at most stages in a game) multiple possible outcomes need to be retained to compare against next possible outcomes. Consequently, this strategy would cause high working memory load.

Besides the question which strategy is preferred (i.e., backward reasoning or forward reasoning plus backtracking), we investigate whether strategy preference can be influenced by task factors. The latter question is inspired by the work of Hedden and Zhang [9]. An important but also criticized aspect of that study was that each participant (assigned to the role of Player 1) was asked to predict the decision of Player 2 first, before making a decision [31]. As this procedure *prompts* perspective taking, ToM reasoning might not have been completely spontaneous [31], [9], [32]. In fact, we have shown that *prompting* participants for predictions indeed has a positive effect on performance [11], [12]. In the current study, we investigate whether prompting may also have an effect on participants’ preferences for any of the strategies.

Because Marble Drop has a predominantly visual interface and both strategies clearly predict a distinct succession in which the payoffs are to be compared, we employed eye tracking to measure the online (i.e., ongoing) process of ToM reasoning. Eye tracking has been used extensively in visual search tasks and reading tasks [33], [34], and in complex visual problem solving tasks [35], [36]. These studies have shown correlations between eye movements, on the one hand, and cognitive processes and higher-level strategies, on the other hand. For example, Kong et al. [35] found a strong correlation between participants’ visual working memory capacity and their eye movements while solving a nontrivial problem-solving task, the traveling salesman problem. Eye tracking has also been proven successful in exposing strategies in another complex (but non-social) reasoning task [36]. Based on the eye movements of participants that played the game of SET, Nyamsuren and Taatgen [36] were able to distinguish between bottom-up visual processes and top-down planning processes. They were also able to detect in-game strategy shifts in participants.

An advantage of eye tracking is that it is an unobtrusive measure; participants were not constrained in any other way than in the original task setting. In contrast, other studies on online ToM reasoning required task modifications that may have influenced participants’ strategies. For example, in Johnson, Camerer, Sen, and Rymon’s computer task [37], participants had to uncover task-relevant information that was hidden behind boxes displayed on the computer screen. The participants had to move the mouse cursor over a box to reveal the information behind it. Consequently, they might have felt disinclined to repeatedly move around the cursor to inspect each box’s content. Tracking the eye movements (with a desk-mounted eye tracker) does not constrain participants so much.

In sum, the literature has identified one optimal strategy (backward reasoning), and we propose another (forward reasoning plus backtracking). Both strategies are clearly distinct from each other. This study aims to identify which strategy explains participants’ performance in a ToM task best. It also investigates whether *prompting* participants for predictions has an effect on their strategies. We use eye tracking because it is an appropriate tool for showing whether the general direction of the eye movements, and thus reasoning, is either forward or backward.

## Methods

### Ethics Statement

The Ethical Committee Psychology (ECP) of the University of Groningen approved this study. Written informed consent as approved by the ECP was obtained from each participant before conducting the experiment.

### Participants

Twenty-three first-year psychology students (14 female) with a mean age of 20.8 years (ranging from 18 to 24 years) participated in exchange for course credit. All participants had normal or corrected-to-normal visual acuity. None of the participants had difficulties distinguishing between the colors (blue and orange) presented in the experiment^1^.

### Stimuli

Instead of using numerical payoffs, which are commonly used in strategic games, we chose for colored marbles to counter numerical but non-optimal strategies such as, for example, minimizing the opponent’s outcomes, or maximizing the difference in Player 1 and Player 2 outcomes.

#### Payoffs

The payoffs were marbles of 4 different shades that could be ordered from light to dark. The colors of the marbles were shades of orange and blue, taken from the HSV (i.e., hue, saturation and value) space. A sequential color palette was computed by varying saturation, for a given hue and value. This resulted in 4 shades (with saturation from.2 to 1) for both of the colors orange (hue = .1, value = 1) and blue (hue = .6, value = 1). The participants did not have any difficulties distinguishing between the shades of either color.

#### Payoff structures

The payoff structure (i.e., configuration of payoffs) and strategy preference determine the complexity of the reasoning required of Player 1, the participant. For example, a forward reasoning Player 1 immediately knows what to do if payoff-pair A contains his darkest marble: stop the game (i.e., remove the left-side trapdoor). In this case, Player 1 does not have to reason about Player 2′s reasoning about Player 1. Therefore, we excluded this payoff structure, as it cannot inform us about second-order ToM. We only selected payoff structures that required Player 1 to reason about the decision at each of the three decision points (i.e., sets of trapdoors).

In line with Hedden and Zhang’s criteria [9], we considered payoff structures to be diagnostic of second-order ToM reasoning if, at the first set of trapdoors, second-order reasoning yielded a decision opposite to a decision based on first-order ToM reasoning. The payoff structures were balanced for the number of correct decisions to remove the left/right trapdoor, for both Player 1 and Player 2. The payoff structures are provided in Material S1.

### Design

The experiment consisted of three blocks: a training block and two test blocks. The training block was meant to familiarize participants with the rules of Marble Drop. In the first test block we manipulated whether participants were prompted to predict Player 2′s decision. The first test block was followed by a second one, in which none of the participants had to make predictions anymore. This block was meant to measure the longevity of the effect of prompting participants for predictions.

### Procedure

Participants were seated in front of a 20-inch computer monitor, at 70 cm distance. An Eyelink 1000 eye-tracker was used to record the eye movements of the dominant eye, at a sample-rate of 500 Hz. The eye tracker was calibrated to each participant’s dominant eye. Participants were always assigned to the role of Player 1. The target color, either blue or orange (marbles and trapdoors), was counterbalanced between participants. Participants were instructed that their goal was to maximize their payoffs, that is, to attain the darkest possible marble of their target color. Participants were told truthfully that they were playing against a computer-simulated Player 2^2^, whose goal was to maximize its payoffs. Participants were also instructed that the computer was programmed to look ahead and take into account the participant’s last possible decision (i.e., Player 1′s decision at the last set of trapdoors).

In the training block, participants were presented with 20 games of increasing difficulty. To familiarize the participants with the setup of the Marble Drop games, participants were first presented four trivial two-bin games that did not require ToM reasoning (Figure 1a). These two-bin games were followed by a set of eight three-bin games (Figure 1b), and a set of eight four-bin games (Figure 1c). The three-bin games require first-order ToM, because the participants have to reason about the decision of Player 2 at the second decision point (i.e., set of trapdoors). As discussed earlier, the four-bin games require second-order ToM. Each training game was played until either the participant or the computer decided to stop the game, by removing the left-side trapdoor, or until the last possible decision was made. After each game, participants were presented feedback displaying either “correct” if they obtained the darkest possible marble, or “incorrect” if they failed to do so. The feedback never indicated why a response was incorrect. Thus, participants had to find out themselves why an incorrect decision was incongruent with the other player’s mental state. As the participants’ performance on the eight four-bin games is indicative of their pre-experimental level of second-order ToM reasoning, we have included these items in the analyses.

*Prompting* participants for predictions was manipulated in the first test block, which consisted solely of second-order games. Participants were randomly assigned to either the so-called *Prompt* group (10 participants), or the so-called *No-Prompt* group (13 participants). Participants in the *Prompt* group were asked to enter their prediction of Player 2′s decision at the second decision point before they were asked to enter their own decision at the first decision point. Participants in the *No-prompt* group were not explicitly asked to make any predictions. In this block, games stopped immediately after entering a decision. Feedback was presented after entering a prediction, if a prediction was queried, and after entering a decision. Feedback mentioned only whether a response was (in)correct. The first test block consisted of 32 trials; each of the 16 payoff structures was presented twice. The order was randomized.

The second test block was similar to the first one except that none of the participants were explicitly queried for a prediction anymore. This block also consisted of 32 trials.

## Results and Discussion

### Behavioral Results

**Figure 2:**
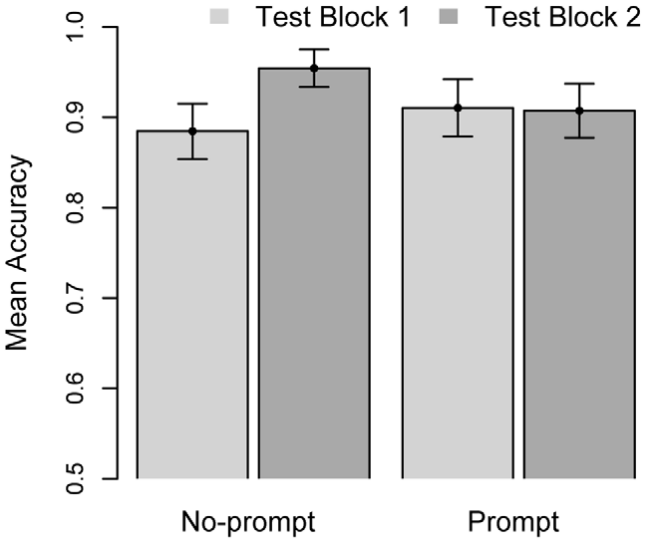
Mean accuracy in *No-prompt* and *Prompt* conditions, depicted separately for test blocks 1 and 2. Error bars represent standard errors.

Figure 2 depicts the mean accuracy of participants playing second-order Marble Drop games. The mean accuracy scores were analyzed by means of repeated-measures ANOVA. However, the scores were first arcsine-transformed to preserve homogeneity of variance. The analysis included the between-subjects factor *prompting* (*No-prompt/Prompt*) and the within-subjects factor *block* (*Test Block 1/Test Block 2*).

In contrast to our earlier work [12], the factor *prompting* was not significant, F(1, 21) = .1, *ns*. On average, asking participants to predict Player 2′s decision did not (positively) influence their performance. The lack of an overall effect of *prompting* might have been due to ceiling effects, as the mean accuracy was very high, around 90% in both test blocks.

The interaction between *prompting* and *block* was significant: F(1, 21) = 4.61, p = .044. On average, accuracy increased from Test Block 1 to Test Block 2, F(1, 21) = 5.09, *p* = .035, but that effect was mainly due to increasing accuracy in the *No-prompt* group. A possible explanation for the interaction might be that participants in the *Prompt* group, in contrast to participants in the *No-prompt* group, had to adjust to an experimental procedure that changed with each subsequent test block. This could have hindered their performance, which did not significantly differ between the two test blocks, t(9) = .12, *ns*.

### Eye Tracking Results

**Figure 3:**
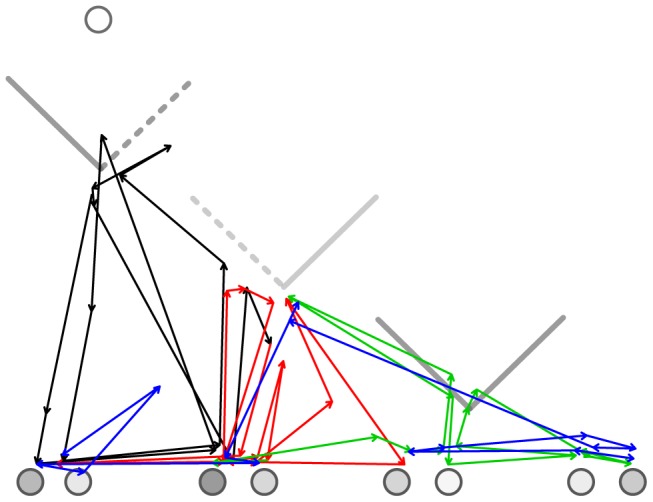
Example of a participant’s fixations in a particular game. The succession of fixations is indicated by arrows, which are superimposed on the payoffs and trapdoors (i.e., decision points). The first 15 fixations are depicted in black, fixations 16–30 in red, fixations 31–45 in green, and fixations 46–61 are depicted in blue. The succession of fixations on payoffs and trapdoors seems to indicate forward reasoning, followed by backtracking, which is indicated by the blue arrows that eventually go back to the first payoff pair.

Eye movements were measured to distinguish between the strategies that participants may have used in second-order Marble Drop games, as *backward* and *forward reasoning* would clearly yield distinctive successions of fixations on each player’s payoffs. The default parameters of the Eyelink 1000 eye tracker were used to extract fixations from the eye movement data. Figure 3 gives an example of a participant’s succession of fixations in a particular game.

Each pair of payoffs was considered to be an area of interest (AOI). However, we did not define fixed AOIs with specific x and y coordinates. As the AOIs corresponding with the payoff-pairs are relatively small, a slightly inaccurate calibration of the eye tracker to a participant’s dominant eye would shift his or her fixations outside of the AOIs. Therefore, cluster analysis was used to find four clusters of fixations in each participant’s dataset, each cluster corresponding to a payoff-pair. The clustering algorithm used was a more robust version of k-means clustering [38]. Fixations in the first (i.e., leftmost) cluster were labeled with the letter *A*; fixations in the second cluster were labeled with the letter B, and so forth. The labels are depicted above the payoff-pairs in Figure 1c. All following analyses solely include fixations that fall within these AOIs.

#### Onset times of fixations on payoff-pairs

**Figure 4:**
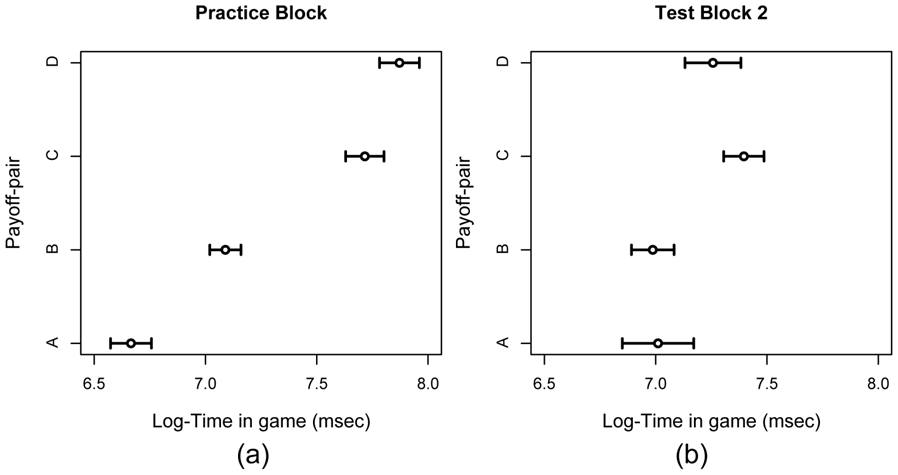
The logarithm of the onset times (in msec) of fixations on each payoff-pair. The onset times are depicted separately for the practice block (a) and Test Block 2 (b). The error bars represent standard errors.

We analyzed the in-game times at which each cluster (i.e., payoff-pair) was first fixated, as these so-called onset times may indicate a general direction of reasoning in second-order Marble Drop games. The onset times were averaged across trials, separately for each participant (i.e., the 8 second-order trials from the practice block, and 32 trials from test block 2). The onset times were log-transformed, because their distribution was skewed to the right. The mean onset times (across participants) are depicted in Figure 4. We collapsed the data across the *Prompt* group and the *No-prompt* group, as there were no significant differences between these groups.

Figure 4a shows monotonically increasing onset times in the practice block, which indicates a forward (i.e., left-to-right) general direction of reasoning. All pairwise comparisons are significant, AB: *p*<.001; AC: *p*<.001; AD: *p*<.001; BC: *p* = <.001; BD: *p*<.001; CD: *p* = .028. The p-values are corrected by means of the Bonferroni-Holm method [39] to account for family-wise error rate.

Presumably, participants’ strategies were most stable near the end of the experiment. However, the timing of the first fixations on each payoff-pair does not inform us on what these strategies might have been (see Figure 4b). The onset times do not increase monotonically anymore, in contrast to the onset times in the practice block. However, payoff-pairs A and B are still fixated earlier than payoff-pairs C and D. The average difference in onset times is significant, t(45) = −2.76, p = .008.

As the onset times do not strongly correspond with either one of the candidate strategies, we analyzed the entire fixation sequences, which might reveal patterns corresponding to backward and/or forward reasoning.

#### Fixation sequences

Before presenting the statistics on the entire fixation sequences, we will first explain the statistical procedure, which involves several steps.

**Figure 5:**
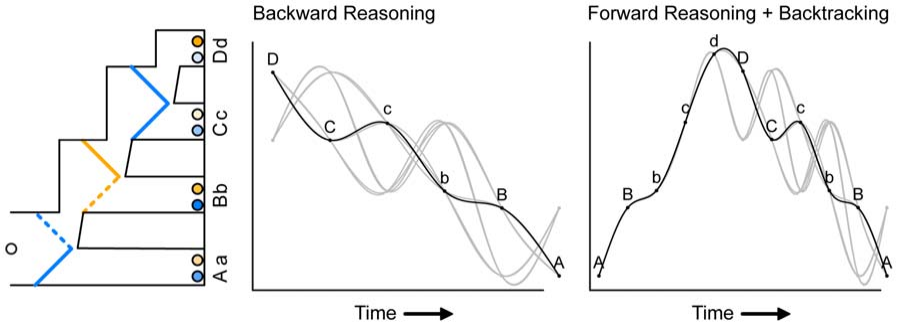
An example second-order Marble Drop game (left panel), and associated fixation sequences predicted on the basis of backward reasoning (middle panel) and forward reasoning plus backtracking (right panel). The fixation sequences represented by the black lines are annotated for AOI (A – D; a – d), and those represented by the grey lines are not. Player 1′s payoffs are labeled with uppercase A, B, C, and D. Player 2′s payoffs are labeled with lowercase a, b, c, and d. The sequences are depicted on “eye movement paths” for illustrative purposes.

For each game, we predicted which payoffs would be fixated, and in which succession, given a particular strategy. The left panel of Figure 5 depicts an example game, the middle panel depicts fixation sequences that were predicted on the basis of backward reasoning, and the right panel depicts fixation sequences that were predicted on the basis of forward reasoning plus backtracking. For illustrative purposes, fixations on Player 2′s marbles were labeled with lowercase letters a, b, c, and d, and fixations on Player 1′s marbles with uppercase letters A, B, C, and D. Each line in the last two panels of Figure 5 represents a possible sequence of fixations given the corresponding strategy.

Backward reasoning yields eight possible fixation sequences for each individual game. Namely, a comparison between two payoffs can yield two possible successions of fixations, for example <D, C> versus <C, D>, and there are three comparisons to be made when applying backward reasoning. Thus, there is a total of two to the power of three, which is eight, possible fixation sequences. We granted forward reasoning plus backtracking the same degrees of freedom by applying the same procedure to the backtracking part, which is essentially the same as backward reasoning.

Given that we predicted fixations on individual marbles, we had to label each observed fixation for the specific marble that was fixated. We used cluster analyses to find two sub-clusters within each of the previously found payoff-pair clusters. Each left-side sub-cluster was considered to contain fixations on Player 1′s marbles, and each right-side sub-cluster was considered to contain fixations on Player 2′s marbles.

It is important to note that our implementations of the two strategies are idealizations, as we did not implement cognitive constraints such as, for example, working memory capacity. Consequently, the *predicted* fixation sequences did not contain repetitions. In contrast, the *observed* fixations sequences *did* contain repetitions, as participants would re-fixate payoffs if they had forgotten previously attended payoffs and comparisons. Figure 3 clearly shows an example of a participant repeatedly fixating payoffs. We accounted for these memory effects by collapsing repeating patterns in the observed fixation sequences. For example, both ***AA*** *aBbCd* and ***AaAa*** *BbCd* would collapse to *AaBbCd*.

To evaluate how closely our predicted fixation sequences match the observed fixation sequences, we calculated the Levenshtein distance, which is the minimal number of insertions, deletions, and substitutions to get from one sequence to another. For example, if an observed fixation sequence for the game in Figure 5 would consist of AOIs <D, ***d*** **,** C, c, b, B, A>, we would find strong evidence in favor of backward reasoning, as it differs only one fixation (i.e., ***d***) from one of the predicted sequences of AOIs <D, C, c, b, B, A>. Importantly, the observed fixation sequence is compared with a set of eight predicted fixation sequences, thus eight Levenshtein distances are calculated, and the minimum Levenshtein distance is taken. To account for varying lengths of observed and predicted fixation sequences, the Levenshtein distance is normalized by dividing it by the length of whichever of the two sequences is longer, either the observed or the predicted one.

**Figure 6:**
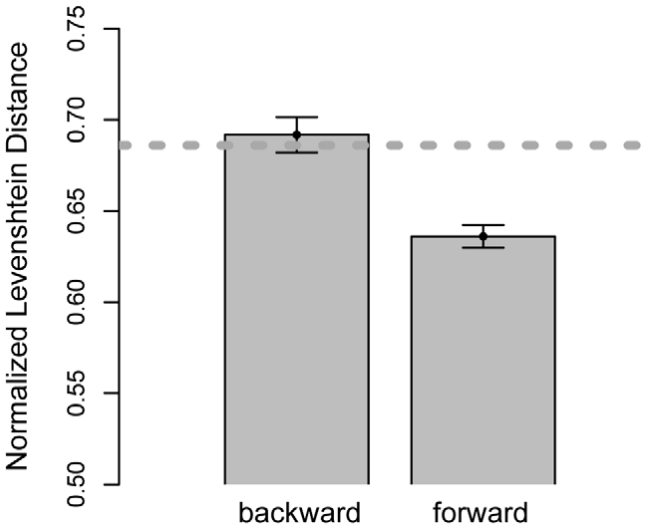
The average normalized Levenshtein distance between the observed sequence, on the one hand, and the closest of the set of predicted sequences, on the other hand. The dotted line is considered a baseline measure, which is the average normalized Levenshtein distance between an observed sequence and its randomized version. The error bars represent standard errors.

According to the procedure described above, the normalized Levenshtein distance was calculated for each individual trial (i.e., 32 trials per participant per test block). The normalized Levenshtein distance was averaged across trials, separately for each participant. Figure 6 depicts the mean normalized Levenshtein distance in Test Block 2, in which strategy preference is most stable.

We collapsed the data across the *No-prompt* group and the *Prompt* group, as the eye movement patterns did not significantly differ between these groups. Both the main effect of *prompting* and the interaction between *strategy* and *prompting* were not significant, F(1, 21) = .46, *ns*, and F(1, 21) = .71, *ns*, respectively. There are two possible explanations for this: Either *prompting* participants for predictions did not affect their strategy preference, or participants in the *No-prompt* group developed similar strategies on their own.

Figure 6 shows that, on average, the observed fixation sequences are most similar to the fixation sequences predicted on the basis of forward reasoning plus backtracking. The normalized Levenshtein distance is significantly larger for predictions based on backward reasoning, t(22) = 5.64, p<0.001. Figure 6 also depicts a baseline measure (dotted line), which is the average normalized Levenshtein distance between observed fixation sequences, on the one hand, and each sequence randomized, on the other hand. Randomized sequences contain the same frequency of fixations as their observed counterparts, but nevertheless, forward reasoning plus backtracking fits the observed behavior significantly better than the baseline measure, t(22) = 4.91, p<0.001.

#### Sub-patterns in the fixation sequences

To get a better idea of which specific components of the hypothesized strategies describe participants’ reasoning best, we performed exploratory statistics on sub-patterns in the fixation data. The analysis concerns the fixation data from Test Block 2, as participants’ strategies are assumed to be most stable in that test block. We will first describe the procedure of extracting sub-patterns from the fixation sequences, and then provide the results.

We analyzed sub-patterns of three subsequent fixations, as three is the minimal number of fixations that makes a pattern informative of either a forward or backward succession of comparisons between marbles. For example, subsequent fixations on payoff-pairs C, D, and B unambiguously indicate a backward succession of comparisons, even though the first two fixations seem to indicate a forward succession.

All subsequent triplets of fixations were extracted from each individual fixation sequence. If, for example, a trial consisted of fixations on payoff-pairs CDBCAB, sub-patterns CDB, DBC, BCA, and CAB were extracted. We considered fixations on payoff-pairs instead of fixations on individual payoffs (e.g., C versus c), as the latter would yield too many combinations with very low frequencies.

The results of the analyses are presented in Table 1, which shows the 50% most frequent *forward* and *backward* triplets. As can be seen in Table 1, the 50% most frequent triplets contain as many forward as backward triplets, and the frequencies of these triplets are quite similar. This seems to imply that, on average, participants made as many forward as backward comparisons between marbles.

**Table 1:** The 50% most frequent forward and backward fixation triplets.

	Triplet	Proportion
Forward triplets	BCD	0.093
	ABC	0.058
	BDC	0.031
	ABD	0.024
	ACD	0.019
Backward triplets	DCB	0.079
	CDB	0.055
	CBA	0.047
	DCA	0.042
	DBC	0.022

The frequency of each triplet was divided by the total number of triplets, n = 6126, yielding the proportions given in the last column.

**Figure 7:**
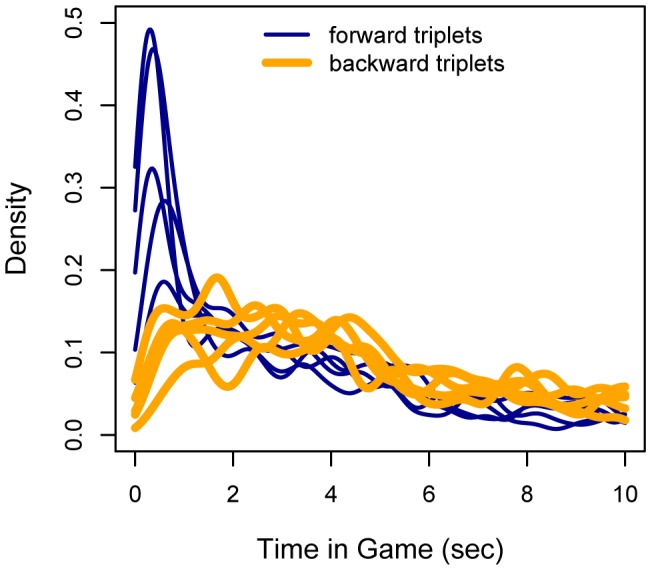
Densities of onset times of *forward* (blue) and *backward* (orange) triplets.

We also analyzed the (in-game) onset times of forward and backward triplets, as these help us to determine whether forward and backward comparisons were made alternately, or forward comparisons first, followed by backward comparisons. Figure 7 depicts the relative likelihood (or probability density function) of onset times of all the triplets, and thus the likelihood of observing a particular triplet at a particular time in a game. Figure 7 clearly shows that all forward triplets have a relatively high likelihood of being observed early in a game, between zero and two seconds, whereas the highest likelihood of observing backward triplets is distributed over the entire range of 0 to 5 seconds.

Backward triplets correspond with either backward reasoning or the backtracking part of forward reasoning, depending on onset time. Early onset times would indicate backward reasoning, whereas late onset times would indicate backtracking. Figure 7 clearly shows that the densities of the backward triplets have less prominent peaks than the densities of the forward triplets. The flat likelihood distribution ranging from 0 to 5 seconds seems to imply that, at least in some games, backward reasoning was applied (indicated by early onsets). The finding that after 2 seconds the density functions of forward triplets are similar to those of the backward triplets implies that forward and backward comparisons were made equally often, presumably in alternating sequence. Figure 3 shows an example of such a pattern.

In sum, the densities in Figure 7 correspond best with the forward reasoning plus backtracking strategy. Given that the proportions of forward and backward triplets are quite similar, we can conclude that at early onset times forward triplets are more probable to be observed than backward triplets. In other words, payoffs are most likely to be compared in a forward succession until the last decision point is reached. Thereafter, backtracking takes place if the optimal outcome appears to be available at an earlier decision point. This succession, of forward comparisons followed by backward comparisons can be iterated multiple times until the highest attainable outcome is ascertained.

### General Conclusions

We investigated strategy preference in a ToM task. Therefore, it was crucial that our task was successful at capturing ToM reasoning. Fortunately, mean accuracy was around 90%, close to ceiling, which means that the participants successfully applied (second-order) ToM in a large proportion of the trials (i.e., Marble Drop games).

Eye movements were measured to discern two candidate strategies with opposite general directions of reasoning: *backward reasoning* and *forward reasoning* plus *backtracking*. The onset times of the first fixations on each payoff-pair seem to imply that, in the practice block, participants compared the payoffs in a forward succession. We analyzed the entire fixation sequences in the second test block and found that the forward reasoning plus backtracking strategy described the fixation sequences best. The observed fixation sequences were more similar to the fixation sequences predicted on the basis of forward reasoning plus backtracking than to the fixation sequences predicted on the basis of backward reasoning. Furthermore, by looking at sub-patterns in the fixation data, we found that, early in games, the likelihood of observing forward successions of comparisons between payoffs is higher than the likelihood of observing backward comparisons. These findings suggest that participants were applying forward reasoning, even though backward reasoning is the game-theoretical favorite strategy.

A possible explanation for a stronger preference for forward reasoning plus backtracking might be that backward reasoning requires deep structural knowledge of the task. Fu and Gray [21] argued that in many interactive tasks, experts’ behavior is rather dependent on, or even driven by, surface characteristics. Thus, the strong spatial and temporal structure of our task might have had a role in the adoption of forward reasoning (plus backtracking). Both the task display and the physics in Marble Drop games strengthen the intuitive and chronological direction of progressing decision points and comparing payoffs in a forward succession. Further research is needed to determine to what extent similar, or other, surface features might encourage the adoption of other strategies.

One could argue against forward reasoning by saying that the left-to-right (i.e., forward) fixations on the payoff-pairs merely represent a ‘scanning phase’ in which the payoffs are explored. However, this explanation does not hold since the participants kept fixating on the decision points (i.e., trapdoors) throughout the entire experiment. In fact, the fixations on the payoffs seemed to be interleaved with fixations on the trapdoors. Figure 3 provides an example of this pattern. For scanning purposes only, fixations on trapdoors are unlikely given that the trapdoors did not vary during the entire experiment (whereas the marbles did vary with each game). A more realistic and functional explanation for fixating trapdoors is reasoning, for example, about “*what would happen if the other player opened the left trapdoor”*.

To conclude, ToM reasoning in games such as Marble Drop seems to progress in a forward succession, from causes (or possible decisions) to possible effects. Lacking a deep structural understanding of the logical problems posed in Marble Drop games, participants preferred to use a well-learned strategy, very similar to causal reasoning, even though it was not the most efficient strategy in this context.

## Supporting Information

Material S1 **The payoff structures that were used in the experiment. Player 1′s predictions and decisions are either 0 (i.e., “stop” the game) or 1 (i.e., “continue” the game).**
(DOC)
