# Correction: What Eye Movements Can Tell about Theory of Mind in a Strategic Game

**DOI:** 10.1371/annotation/07d6a9ae-a794-4741-9c47-ef741fcc01fc

**Published:** 2013-06-06

**Authors:** Ben Meijering, Hedderik van Rijn, Niels A. Taatgen, Rineke Verbrugge

There is a sentence missing from the Funding Statement. The following is the missing sentence:

Open access publication of this article was financially supported by NWO's Incentive Fund Open Access Publications (036.002.111).

