# Supplementary material for: What Eye Movements Can Tell about Theory of Mind in a Strategic Game
Source: PLoS One. 2012 Sep 28;7(9):e45961. doi: 10.1371/journal.pone.0045961 (PMC3461025; doi:10.1371/journal.pone.0045961)
Supplement: Material S1 — The payoff structures that were used in the experiment. Player 1′s predictions and decisions are either 0 (i.e., “stop” the game) or 1 (i.e., “continue” the game). (DOC) [file pone.0045961.s001.doc]

# SUPPLEMENTARY MATERIAL S1

Below, we list the payoff structures that were used in the experiment. Player 1’s predictions and decisions are either 0 (i.e., “stop” the game) or 1 (i.e., “continue” the game).

| Payoffs Player 1 | | |  | Payoffs Player 2 | | |  |  |  |
| --- | --- | --- | --- | --- | --- | --- | --- | --- | --- |
| A | B | C | D | A | B | C | D | Prediction | Decision |
| *Two-bin payoff structures* | | | |  |  |  |  |  |  |
| 1 | 3 |  |  | 3 | 2 |  |  |  | 1 |
| 3 | 1 |  |  | 2 | 1 |  |  |  | 0 |
| 4 | 2 |  |  | 2 | 4 |  |  |  | 0 |
| 3 | 4 |  |  | 2 | 4 |  |  |  | 1 |
|  |  |  |  |  |  |  |  |  |  |
| *Three-bin payoff structures* | | | |  |  |  |  |  |  |
| 2 | 1 | 3 |  | 1 | 2 | 3 |  | 1 | 1 |
| 2 | 1 | 3 |  | 3 | 2 | 1 |  | 0 | 0 |
| 2 | 3 | 1 |  | 1 | 2 | 3 |  | 1 | 0 |
| 2 | 3 | 1 |  | 3 | 2 | 1 |  | 0 | 1 |
| 3 | 2 | 4 |  | 2 | 3 | 4 |  | 1 | 1 |
| 3 | 2 | 4 |  | 4 | 3 | 2 |  | 0 | 0 |
| 3 | 4 | 2 |  | 2 | 3 | 4 |  | 1 | 0 |
| 3 | 4 | 2 |  | 4 | 3 | 2 |  | 0 | 1 |
|  |  |  |  |  |  |  |  |  |  |
| *Four-bin (i.e., second-order payoff structures* | | | |  |  |  |  |  |  |
| 3 | 1 | 2 | 4 | 2 | 3 | 4 | 1 | 0 | 0 |
| 3 | 1 | 2 | 4 | 4 | 2 | 3 | 1 | 0 | 0 |
| 3 | 2 | 1 | 4 | 1 | 3 | 4 | 2 | 0 | 0 |
| 3 | 2 | 1 | 4 | 4 | 2 | 3 | 1 | 0 | 0 |
| 3 | 4 | 1 | 2 | 1 | 3 | 2 | 4 | 1 | 0 |
| 3 | 4 | 1 | 2 | 2 | 3 | 1 | 4 | 1 | 0 |
| 3 | 4 | 1 | 2 | 3 | 2 | 1 | 4 | 1 | 0 |
| 3 | 4 | 1 | 2 | 4 | 2 | 1 | 3 | 1 | 0 |
| 3 | 4 | 1 | 2 | 1 | 3 | 4 | 2 | 0 | 1 |
| 3 | 4 | 1 | 2 | 2 | 3 | 4 | 1 | 0 | 1 |
| 3 | 4 | 1 | 2 | 3 | 2 | 4 | 1 | 0 | 1 |
| 3 | 4 | 1 | 2 | 4 | 2 | 3 | 1 | 0 | 1 |
| 3 | 1 | 2 | 4 | 2 | 3 | 1 | 4 | 1 | 1 |
| 3 | 1 | 2 | 4 | 4 | 2 | 1 | 3 | 1 | 1 |
| 3 | 2 | 1 | 4 | 1 | 3 | 2 | 4 | 1 | 1 |
| 3 | 2 | 1 | 4 | 4 | 2 | 1 | 3 | 1 | 1 |
